# Cross-Sectional Associations Between Dietary Antioxidant Vitamins C, E and Carotenoid Intakes and Sarcopenic Indices in Women Aged 18–79 Years

**DOI:** 10.1007/s00223-019-00641-x

**Published:** 2019-12-07

**Authors:** A. A. Welch, A. Jennings, E. Kelaiditi, J. Skinner, C. J. Steves

**Affiliations:** 1 Department of Epidemiology & Public Health, Norwich Medical School, University of East Anglia, Norwich Research Park, Norwich, Norfolk NR4 7TJ UK; 2 Faculty of Sport, Health and Applied Science, St Mary’s University, Waldegrave Road, Twickenham, London, TW1 4SX UK; 3 Department of Twin Research and Genetic Epidemiology, King’s College, London, UK

**Keywords:** Sarcopenia, Diet, Vitamin C, Vitamin E, Carotenes, Vitamin A, Skeletal muscle, Grip strength

## Abstract

The prevalence of sarcopenia is increasing in aging populations, so prevention is critical. Vitamins (A, C, E and carotenoids) modify skeletal muscle via protein and collagen synthesis and anti-inflammatory activities. Previous studies have not investigated intake of these vitamins in relation to sarcopenic indices in both younger and older-aged women. Indices of skeletal muscle mass (as fat-free mass (FFM) relative to body size) were measured using DXA and leg explosive power (LEP) using the Nottingham Power Rig in 2570 women aged 18–79 years. Adjusted measures of skeletal muscle were calculated according to quintiles of vitamin C, E, retinol and carotenoid intake, derived from Food Frequency Questionnaires, after stratification by age. Higher vitamin C intake was associated with significantly higher indices of FFM and LEP, (Q5-Q1 = 2.0–12.8%, *P* < 0.01–0.02). Intakes of total and individual carotenoids were significantly associated with indices of FFM and LEP (Q5-Q1 = 1.0–7.5%). Vitamin E was significantly associated with FFM% and FFM_BMI_ only. In mutually adjusted analysis with vitamin C, total carotene, vitamin E and protein in the model, the strongest associations were with vitamin C. These associations were stronger in younger women (< 65 years). For the first time, our research shows higher dietary intakes of antioxidant vitamins, particularly vitamin C, is associated with higher skeletal muscle mass and power in free-living women. These findings have relevance for the treatment and prevention of frailty and sarcopenia throughout adulthood.

**Electronic supplementary material:**

The online version of this article (10.1007/s00223-019-00641-x) contains supplementary material, which is available to authorized users.

## Background and Aims

Across the world, populations are aging rapidly leading to increased prevalence of sarcopenia and frailty, conditions that also increase the risk of osteoporosis, falls, fractures and mortality, leading to longer and more expensive hospital stays. These conditions also impact adversely on quality of life for individuals [1]. The estimated prevalence of sarcopenia in the community is 1–29% in people over the age of 60 years, which rises to 17.7–87% in those living in assisted or residential or care facilities [1–3]. Frailty is estimated to affect 25% of people over the age of 80 years. We need, therefore, strategies to prevent the gradual age-related decline in skeletal muscle mass and function, or *sarcopenic indices,* that start in middle age to maintain intrinsic capacity in older adults [4].

The established mechanisms of aging of skeletal muscle include increases in circulating cytokines and production of reactive oxygen species (ROS), with age which have detrimental effects on synthesis of protein as well as direct cellular damage of skeletal muscle fibres and DNA [5]. Additionally, there are changes to the proportion, quality and viscoelastic properties of the different types of collagen that form the important structural component of skeletal muscle cells, connective tissues and tendons during aging [6]. As endogenous antioxidant efficiency is reduced with aging, and skeletal muscle generates the greatest quantities of ROS in the body, exogenous antioxidant vitamins have potential importance for skeletal muscle health.

Dietary antioxidant vitamins (A, C, E and carotenoids) are, therefore, promising candidates for the prevention and treatment of age-related loss of mass and function. These vitamins influence skeletal muscle and function through their roles as exogenous antioxidant and anti-inflammatory agents. In addition, vitamin C is involved in collagen and carnitine synthesis and retinol in protein metabolism, collagen formation, and lipid oxidation [7, 8].

To date, dietary treatments for sarcopenia and frailty have focused largely on interventions with protein intake, with or without resistance exercise, which is important for skeletal muscle but the effectiveness of intervention studies in individuals with sarcopenia and frailty have been mixed [9, 10]. As there are no effective pharmacological treatments for sarcopenia, frailty and the loss of skeletal muscle mass and function with age, identifying other dietary factors to prevent or attenuate losses of muscle mass and function in middle and early old age is important.

Whilst limited previous research has studied nutritional intake or blood concentrations of vitamin A, C, E or carotenoids and measures of skeletal muscle mass or function in older-aged populations, none has measured their relative effectiveness in relation to a range of indices of both skeletal mass and function in young as well as in older-aged women [11–19]. Therefore, the purpose of this study was to first, understand the associations between: (i) dietary vitamins C, E and A and (ii) the full range of dietary carotenoids; α-carotene, β-carotene, β-cryptoxanthin, lycopene, lutein and zeaxanthin and indices of skeletal muscle mass [i.e. Fat Free Mass Index (FFMI), percentage fat-free mass (FFM%) and fat-free mass adjusted for body mass index (FFM_BMI_)], and function [i.e. hand grip strength, arm muscle quality and leg explosive power (LEP)] in a population of women with a wide age range. Second, to understand whether these associations differed in young women compared with those over the age of 65 years, third to estimate the relationship between the inflammatory cytokine C-reactive protein (CRP) and indices of skeletal muscle health and fourthly, to determine the relative associations of vitamins A, C, E, total carotene and protein by including all the nutrients in the same statistical models to determine which of the vitamins was the most strongly associated with the indices of skeletal muscle health.

## Methods

The TwinsUK registry is an ongoing population study of approximately 12,000 male and female (83%) twins, aged 18–103 years who are representative of singleton populations in the United Kingdom [20]. Data were used from 2570 women who had completed a food frequency questionnaire (FFQ) and attended for dual-energy X-ray absorptiometry (DXA) measurements between 1996 and 2000. Within this group, there were 1914 individuals with measures of leg explosive power and 1658 individuals with measures of high sensitivity C-reactive protein (hs-CRP). Between 2005 and 2008, 949 women completed an FFQ and had grip strength measured (including 512 individuals from the first cohort) (Supplementary Fig. 1). Ethical approval was obtained from St. Thomas’s Hospital Research Ethics Committee and informed consent was acquired from all participants.

**Fig. 1:**
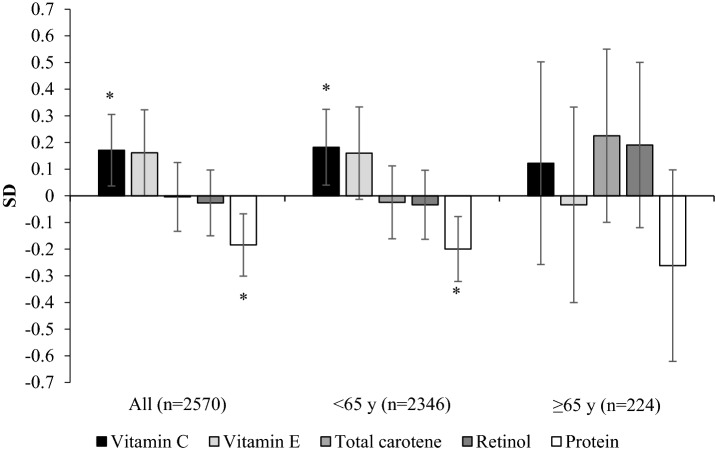
The relative associations of vitamin C, vitamin E, retinol, carotene and protein with percentage fat-free mass in 2570 females aged 18–79 years, stratified by age^1^. Values represent the difference in standardised values of percentage fat-free mass between participants in Q5 vs Q1 of intake (T3-T1 for > 65 years sub-group) with all nutrients included in the model. Values were also adjusted for age, physical activity, smoking status, energy intake and underreporting. * *P*-trend < 0.05

## Dietary Intake

Participants completed a 131 item-validated FFQ with nutrient values calculated using the UK national nutrient database and a database of the carotenoid composition of foods [21, 22]. Individuals were excluded from the dietary analyses if answers to > 10 food items were left blank or the ratio of estimated total energy intake to the estimated basal metabolic rate fell 2 SDs outside the mean ratio. Energy-reporting quality was estimated using the ratio of reported energy intake (EI) to estimated energy expenditure (EER), the EI:EER ratio expressed as the percentage of EI to EER, was calculated and was included as a covariate for adjustment in the statistical analyses [23].

## Muscle Mass, Strength and Power

Fat-free mass (FFM) was measured by DXA scans (Hologic QDR-2000 DXA scanner, Hologic Inc., Waltham, MA, USA) and adjusted to body size using the following calculations: FFMI: FFM (kg)/ height (m^2^) [1], FFM%: FFM (kg)/ weight (kg) *100 and FFM_BMI_: FFM (kg)/ BMI (kg/m^2^). FFM (kg) divided by BMI (kg/m^2^) (FFM_BMI_) takes into account the increase in body size, scaled for height and adjusting FFM for BMI is likely to set meaningful cut-off points for clinical use [1]. Isometric grip strength was assessed using a Jamar hand grip dynamometer (Sammons, Preston, UK) on the dominant arm with reproducibility assessed by repeated measurement on 24 individuals (CV of 11.4%) [24] and adjusted for mean arm lean mass as arm muscle quality (grip strength (kg)/ mean arm lean mass (kg). LEP was measured using the Nottingham Power Rig which assesses the force and velocity of muscle contraction from the quadriceps, normalised for body weight (LEP (watts)/weight (kg)) [25]. LEP is validated with high reliability (reliability coefficient 0.97, coefficient of variation 9.4%, over one week period in adults) [25].

## C-reactive Protein

Circulating hs-CRP was measured by a highly sensitive automated microparticle capture enzyme immunoassay, standardised on the World Health Organisation International Reference Standard for CRP immunoassay as previously described [26].

## Covariates

Information on lifestyle, medication use, menopausal status, and demographic variables were obtained using standardised nurse-administered questionnaires. Weight and height were measured to the nearest 0.1 kg and to the nearest 0.5 cm, respectively. BMI was calculated as weight in kilograms (kg) divided by height in meters squared (m^2^). Leisure- and work-time physical activity was self-reported using a questionnaire significantly correlated with in depth assessments of physical activity in a subset of this cohort [27]. For participants in the grip strength group (*n* = 949 women) who had missing data on physical activity (*N* = 10), the General Practice Physical Activity Questionnaire (GPPAQ) was used. This was based on the original physical activity index developed by the European Prospective Investigation into Cancer and Nutrition (EPIC) cohort. The EPIC physical activity index was previously validated against heart rate monitoring in two independent studies [28].

## Statistical Analyses

Descriptive statistics (means ± SDs or % (n)) were analysed for all participants and for those under 65 years (*n* = 2346) and ≥ 65 years (*n* = 224). Multivariate regression analysis was used to calculate statistical trends and adjusted values (least square means) for FFM%, FFMI, FFM_BMI_, LEP, arm muscle quality and hs-CRP (outcomes) classified by quintile of dietary vitamins C, E and A and dietary carotenoids; α-carotene, β-carotene, β-cryptoxanthin, lycopene, lutein and zeaxanthin (exposures). The percentage difference between quintiles 1 and 5 was calculated as a percentage of the value in quintile 1. As physical activity and smoking habit were related to indices of skeletal muscle the analyses were adjusted for physical activity and smoking habit. All models were adjusted for age (years), physical activity (active, moderately active, inactive), smoking status (never, former, current), energy intake (kcal/d) and potential mis-reporting of energy intake (EI:EER). FFMI was adjusted for age (years), physical activity (active, moderately active, inactive), smoking status (never, former, current), energy intake (kcal/d), potential mis-reporting of energy intake (EI:EER) and fat mass (kg). LEP and arm muscle quality were adjusted for age (years), physical activity (active, moderately active, inactive), smoking status (never, former, current), energy intake (kcal/d), potential mis-reporting of energy intake (EI:EER) and menopausal status (pre-menopausal/ post-menopausal), use of HRT (yes/no) and height (m). High sensitivity (hs) CRP was adjusted for age (years), physical activity (active, moderately active, inactive), smoking status (never, former, current), energy intake (kcal/d), potential mis-reporting of energy intake (EI:EER), and BMI, use of anti-inflammatory medications (yes/no) and HRT (yes/no). Values for hs-CRP were skewed and therefore, natural log-transformed values were used for the analyses.

In analysis stratified by age, we also assessed the associations of FFM%, FFMI, FFM_BMI_ and LEP with quintiles of intake for those under 65 years (*n* = 2346) and tertiles for those ≥ 65 years (*n* = 224). The data for those ≥ 65 years was analysed for tertiles due to the smaller number of women in this group. For these and the comparative analyses we only considered vitamins A, C, E, total carotene and protein intakes as these demonstrated the greatest associations, as shown in Table 2. In additional analyses, to compare the relative associations of vitamins A, C, E, total carotene and protein we included all these nutrients in the same statistical models and standardised the values for FFM%, FFMI, FFM_BMI_ and LEP using Z-scores. Values for the FFM indices and LEP were also adjusted for covariates as described previously, and as in the models shown in Table 3.

Values in the text are means ± SE. A *P* value < 0.05 was considered statistically significant. *P* trend was calculated from the multivariable regression across quintiles of intake of the nutrients. All analyses were performed with Stata statistical software version 14.0 (Stata Corp, College Station, TX) and included the robust cluster regression option in STATA to account for clustering within twin pairs.

## Results

The characteristics of the participants are presented in Table 1, stratified by age (< 65 years and ≥ 65 years). The mean age (± SD) of participants was 48.37 ± 12.7 years and mean (± SD) BMI was 24.9 ± 4.14 kg/m^2^. More than one-half of the participants were moderately active (53.9%), and 18.2% were current smokers. In multivariable analyses, women in the highest quintile of vitamin C intake had signficantly higher FFM%, FFMI, FFM_BMI_ and LEP, compared to those in the lowest quintile (Table 2). Specifically, FFM% was 1.4% higher (± 0.4 *P*-trend < 0.001), FFMI was 0.4 kg/m^2^ higher (± 0.1 *P*-trend = 0.002), FFM_BMI_ was 0.03 kg/kg/m^2^ higher (± 0.01 *P*-trend = 0.023) and LEP was 10.9 w/kg higher (± 2.6 *P*-trend < 0.001), when comparing extreme intake quintiles. In terms of vitamin E, FFM% (Q5-Q1 1.6% ± 0.5 *P*-trend = 0.002) and FFM_BMI_ (Q5-Q1 0.06 ± 0.02 *P*-trend = 0.002) were positively associated with intakes. For total carotene, associations were also significant for FFM% (Q5-Q1 0.6% ± 0.4 *P*-trend = 0.028), FFMI (Q5-Q1 0.2 ± 0.1 *P*-trend = 0.012) and LEP (Q5-Q1 6.4 ± 2.7 *P*-trend = 0.011). The results of the associations between the nutrients and arm muscle quality were non-significant ranging from a beta coefficient (95% CI) of − 0.075 (− 0.199, 0.048) per quintile for vitamin C intake (*P*-trend = 0.231) to 0.036 (− 0.150, 0.222) per quintile for vitamin E intake (*P*-trend = 0.705). The results of the associations between the nutrients and hs-CRP were also non-significant ranging from a beta coefficient (95% CI) of − 0.008 (− 0.046, 0.029) per quintile for vitamin C intake (*P*-trend = 0.666) to − 0.023 (− 0.070, 0.025) per quintile for vitamin E intake (*P*-trend = 0.349). Also no associations were observed for vitamin A (measured as retinol).

**Table 1:** Characteristics and dietary intakes of *n* = 2570 females aged 18–79 years, stratified by age^a^

Characteristic	All	< 65 years	≥ 65 years
*n* =	2570	2346	224
Age (years)	48.3 ± 12.7	46.5 ± 11.6	68.0 ± 2.81
BMI (kg/m^2^)	24.9 ± 4.14	24.8 ± 4.11	26.1 ± 4.23
Fat mass (kg)	22.7 ± 7.87	22.5 ± 7.90	25.0 ± 7.26
Fat free mass (%)	61.1 ± 6.50	61.4 ± 6.46	58.0 ± 6.08
Fat Free Mass Index (kg/m^2^)	15.0 ± 1.72	15.0 ± 1.71	15.0 ± 1.80
Fat-free mass/ BMI (kg/ kg/m^2^)	1.62 ± 0.23	1.63 ± 0.23	1.47 ± 0.19
Leg explosive power^b^ (watts)	89.8 ± 36.8	91.3 ± 36.7	67.5 ± 30.8
Leg explosive power^b^ (watts/kg)	90.9 ± 36.5	92.4 ± 36.3	68.3 ± 31.6
hs-CRP^c^ (mg/L)	2.49 ± 2.30	2.47 ± 2.30	2.72 ± 2.26
Grip strength^d^ (kg)	28.8 ± 5.95	30.1 ± 5.66	25.3 ± 5.20
Energy intake (kcal/d)	1979 ± 524	1978 ± 533	1989 ± 415
Protein (% energy)	16.6 ± 2.62	16.6 ± 2.62	16.9 ± 2.68
Vitamin C (mg/d)	155 ± 80.2	154 ± 80.4	165 ± 78.3
Vitamin E (mg/d)	11.4 ± 4.57	11.3 ± 4.60	11.7 ± 4.26
Total carotene (µg/d)	3448 ± 1944	3416 ± 1964	3777 ± 1688
*α*-Carotene (µg/d)	559 ± 416	554 ± 418	621 ± 389
*β*-Carotene (µg/d)	3091 ± 1757	3062 ± 1775	3398 ± 1529
Retinol (µg/d)	559 ± 789	545 ± 793	700 ± 728
*β*-Cryptoxanthin (µg/d)	200 ± 194	201 ± 197	192 ± 166
Lycopene (µg/d)	1347 ± 958	1364 ± 968	1165 ± 820
Lutein + zeaxanthin (µg/d)	2267 ± 1478	2253 ± 1501	2413 ± 1204
Underreporting (EI:EER, %)	87.4 ± 24.6	86.7 ± 24.6	95.0 ± 24.2
Physical activity (active, %)	24.2% (622)	23.9% (561)	27.2% (61)
Moderately active (%)	53.9% (1385)	54.2% (1271)	50.9% (114)
Inactive (%)	21.9% (563)	21.9% (514)	21.9% (49)
Smoking status (current, %)	18.2% (468)	19.1% (449)	8.48% (19)
Menopausal status (post-menopausal, %)	47.4% (1218)	42.5% (997)	98.7% (221)
Anti-inflammatory medication^c^ (yes, %)	6.15% (102)	6.06% (90)	6.98% (12)
Hormone replacement therapy^c^ (yes, %)	6.33% (105)	6.33% (94)	6.40% (11)

*EI:EER* ratio of reported energy intake to estimated energy requirements

^a^Values are mean ± SD or % (*n*), *n* = 2570

Values for a subset of ^b^1914

^c^1658

^d^949 participants

**Table 2:** Fat free mass indices and leg explosive power by quintile of nutrient intake in 2570 females aged 18–79 years^a^

	Intake^b^	FFM (%)	FFM/BMI	FFMI (kg/m^b^)	LEP^c^ (w/kg)
Vitamin C (mg/d)					
Q1	68.8 ± 16.4	60.4 (59.8,60.9)	1.60 (1.58,1.62)	14.8 (14.7,15.0)	85.2 (81.8,88.6)
Q2	108 ± 9.43	60.9 (60.4,61.4)	1.61 (1.59,1.63)	15.0 (14.9,15.2)	90.8 (87.2,94.4)
Q3	141 ± 10.1	61.1 (60.6,61.6)	1.62 (1.60,1.64)	15.0 (14.9,15.1)	90.1 (86.4,93.8)
Q4	181 ± 13.8	61.3 (60.8,61.8)	1.62 (1.60,1.64)	15.1 (14.9,15.2)	92.0 (88.2,95.9)
Q5	276 ± 80.3	61.8 (61.3,62.3)	1.63 (1.61,1.65)	15.2 (15.1,15.3)	96.1 (92.3,99.9)
*P*-trend	–	< 0.01	0.02	< 0.01	< 0.01
Q5-Q1%	–	2.36	1.99	2.40	12.79
Vitamin E (mg/d)					
Q1	5.90 ± 1.16	60.3 (59.7,60.9)	1.58 (1.56,1.61)	14.9 (14.8,15.1)	89.2 (84.8,93.6)
Q2	8.59 ± 0.63	60.7 (60.2,61.2)	1.60 (1.58,1.62)	15.1 (14.9,15.2)	90.6 (86.9,94.3)
Q3	10.7 ± 0.64	61.3 (60.8,61.8)	1.63 (1.61,1.65)	15.1 (14.9,15.2)	91.9 (88.1,95.8)
Q4	13.3 ± 0.81	61.2 (60.7,61.8)	1.62 (1.60,1.64)	15.0 (14.9,15.2)	90.9 (87.3,94.5)
Q5	18.3 ± 3.39	61.9 (61.2,62.5)	1.64 (1.62,1.66)	15.0 (14.8,15.2)	91.6 (87.1,96.2)
*P*-trend	–	< 0.01	< 0.01	0.70	0.54
Q5-Q1%	–	2.60	3.54	0.42	2.77
Total carotene (µg/d)					
Q1	1342 ± 377	60.8 (60.3,61.3)	1.61 (1.59,1.63)	14.9 (14.7,15.0)	85.6 (81.8,89.3)
Q2	2379 ± 320	60.9 (60.4,61.4)	1.61 (1.59,1.63)	15.0 (14.9,15.1)	91.2 (87.7,94.8)
Q3	3223 ± 189	60.8 (60.3,61.3)	1.61 (1.59,1.63)	15.0 (14.9,15.2)	90.8 (87.0,94.5)
Q4	3991 ± 321	61.6 (61.1,62.1)	1.63 (1.61,1.65)	15.1 (15.0,15.3)	94.8 (91.1,98.4)
Q5	6303 ± 2107	61.4 (60.9,62.0)	1.62 (1.60,1.64)	15.1 (14.9,15.2)	91.9 (88.2,95.7)
*P*-trend	–	0.03	0.11	0.01	0.01
Q5-Q1%	–	1.03	0.96	1.44	7.45
*α*-Carotene (µg/d)					
Q1	131 ± 62.8	61.1 (60.6,61.7)	1.62 (1.60,1.64)	14.9 (14.8,15.0)	88.1 (84.6,91.7)
Q2	265 ± 88.2	61.1 (60.6,61.6)	1.62 (1.60,1.64)	15.0 (14.9,15.1)	90.8 (86.9,94.8)
Q3	605 ± 9.02	60.8 (60.3,61.2)	1.60 (1.58,1.62)	15.1 (14.9,15.2)	91.8 (88.3,95.3)
Q4	638 ± 11.7	61.0 (60.5,61.6)	1.62 (1.59,1.64)	15.0 (14.9,15.2)	92.8 (89.2,96.5)
Q5	1158 ± 467	61.4 (60.9,62.0)	1.62 (1.60,1.64)	15.1 (15.0,15.3)	90.7 (86.9,94.4)
*P*-trend	–	0.62	0.94	0.03	0.24
Q5-Q1%	–	− 0.46	− 0.02	− 1.61	2.85
*β*-Carotene (µg/d)					
Q1	1194 ± 344	60.7 (60.2,61.2)	1.60 (1.58,1.63)	14.9 (14.7,15.0)	85.6 (81.8,89.4)
Q2	2139 ± 272	61.0 (60.6,61.5)	1.61 (1.60,1.63)	15.0 (14.9,15.2)	91.4 (87.8,94.9)
Q3	2861 ± 175	60.9 (60.4,61.4)	1.61 (1.59,1.63)	15.0 (14.9,15.1)	90.1 (86.4,93.8)
Q4	3589 ± 294	61.4 (60.9,61.9)	1.62 (1.60,1.64)	15.1 (15.0,15.3)	95.3 (91.6,98.9)
Q5	5673 ± 1911	61.4 (60.9,62.0)	1.62 (1.60,1.65)	15.1 (14.9,15.2)	91.9 (88.2,95.7)
*P*-trend	–	0.05	0.17	0.01	< 0.01
Q5-Q1%	–	1.20	1.23	1.50	7.38
Retinol (µg/d)					
Q1	137 ± 43.1	61.2 (60.7,61.8)	1.61 (1.59,1.63)	15.1 (14.9,15.3)	92.1 (88.1,96.2)
Q2	245 ± 25.1	60.8 (60.3,61.3)	1.60 (1.58,1.62)	15.0 (14.9,15.2)	90.7 (87.0,94.5)
Q3	347 ± 36.6	61.0 (60.5,61.5)	1.61 (1.59,1.63)	15.0 (14.8,15.1)	89.7 (86.2,93.2)
Q4	639 ± 187	61.4 (60.8,61.9)	1.63 (1.60,1.65)	15.1 (15.0,15.3)	90.8 (87.2,94.3)
Q5	1426 ± 1412	61.0 (60.5,61.6)	1.62 (1.61,1.64)	14.9 (14.8,15.1)	91.0 (87.0,95.0)
*P*-trend	–	0.85	0.14	0.23	0.78
Q5-Q1%		− 0.36	0.91	− 1.30	− 1.24
*β*-Cryptoxanthin (µg/d)					
Q1	38.3 ± 16.8	60.7 (60.2,61.2)	1.60 (1.58,1.62)	14.9 (14.8,15.0)	87.7 (84.0,91.4)
Q2	82.9 ± 12.1	61.0 (60.5,61.5)	1.62 (1.60,1.64)	15.0 (14.8,15.1)	89.1 (85.6,92.5)
Q3	146 ± 25.2	61.0 (60.5,61.4)	1.61 (1.59,1.63)	15.1 (15.0,15.2)	90.6 (87.1,94.1)
Q4	234 ± 36.4	61.5 (61.0,62.0)	1.63 (1.61,1.65)	15.0 (14.9,15.2)	93.7 (90.2,97.3)
Q5	499 ± 230	61.4 (60.8,61.9)	1.62 (1.60,1.64)	15.1 (15.0,15.3)	93.2 (89.4,97.0)
*P*-trend	–	0.03	0.22	0.03	< 0.01
Q5-Q1%	–	1.09	0.87	1.50	6.30
Lycopene (µg/d)					
Q1	412 ± 168	61.0 (60.4,61.5)	1.61 (1.59,1.63)	15.0 (14.8,15.1)	87.2 (83.7,90.7)
Q2	821 ± 99.2	61.3 (60.8,61.8)	1.63 (1.61,1.65)	15.0 (14.8,15.1)	91.5 (87.4,95.5)
Q3	1161 ± 100	61.4 (60.9,61.8)	1.63 (1.61,1.65)	15.0 (14.9,15.1)	93.3 (89.6,97.1)
Q4	1573 ± 154	61.2 (60.7,61.6)	1.62 (1.60,1.63)	15.1 (14.9,15.2)	89.8 (86.2,93.4)
Q5	2771 ± 1121	60.6 (60.1,61.2)	1.59 (1.57,1.61)	15.1 (15.0,15.3)	92.5 (88.5,96.5)
*P*-trend	–	0.33	0.05	0.03	0.18
Q5-Q1%	–	− 0.58	− 1.57	1.29	6.05
Lutein + zeaxanthin (µg/d)					
Q1	835 ± 261	60.5 (60.0,61.1)	1.59 (1.57,1.61)	15.0 (14.8,15.1)	88.4 (84.6,92.2)
Q2	1462 ± 153	60.6 (60.2,61.1)	1.60 (1.58,1.62)	15.0 (14.9,15.2)	87.7 (84.4,91.0)
Q3	1970 ± 152	61.1 (60.6,61.6)	1.62 (1.60,1.64)	15.0 (14.9,15.2)	90.3 (86.8,93.8)
Q4	2602 ± 216	61.3 (60.8,61.9)	1.62 (1.61,1.64)	15.1 (14.9,15.2)	93.1 (89.4,96.8)
Q5	4464 ± 1744	61.9 (61.3,62.4)	1.64 (1.62,1.66)	15.0 (14.9,15.2)	94.8 (91.0,98.6)
*P*-trend	–	< 0.01	< 0.01	0.37	< 0.01
Q5-Q1%	–	2.27	3.15	0.52	7.23

*FFM* fat-free mass, *FFMI* Fat Free Mass Index and *LEP* leg explosive power

^a^Values are adjusted means (least square means) ± SE, *n* = 2570. Means were adjusted for age, physical activity, smoking status, energy intake, protein intake and underreporting and Fat Free Mass Index was additionally adjusted for fat mass. Participant numbers by quintile were Q1 = 514; Q2 = 514; Q3 = 514; Q4 = 514; Q5 = 514

^b^Intake values are unadjusted means ± SD

^c^Values are mean (least square means) ± SE, *n* = 1914. Means were adjusted for age, physical activity, smoking status, energy intake, protein intake, underreporting, menopausal status, hormone replacement therapy and height. Participant numbers by quintile were Q1 = 383; Q2 = 383; Q3 = 383; Q4 = 383; Q5 = 382

## Individual Carotenoids

Intakes of all the individual carotenoids, with the exception of leutein + zeaxanthin, were significantly associated with FFMI. The strongest association was observed for *α*-carotene intake (Q5-Q1 0.24 kg/m^2^ ± 0.1 *P*-trend = 0.03, 1.6%). Significant associations were also found with FFM% for ß-cryptoxthanin and with FFM% and FFM_BMI_ for lutein and zeaxthanin, with interquintle differences ranging from 1.1 to 7.2%. LEP was associated significantly with the carotenoids, with the exception of α-carotene, with differences ranging from 6.3 to 7.5% when comparing extreme quintiles of intake.

## Age-Stratified Analyses

In age-stratified analyses, we observed that associations between vitamin C and E with FFM% were observed in the younger (< 65 years) but not the older (≥ 65 years) subset (Table 3). Specifically, vitamin C was associated with a 0.3% (95% CI 0.1, 0.5 *P*-trend = 0.002) and vitamin E with a 0.4% (95% CI 0.2, 0.7 *P*-trend = 0.001) higher FFM% per quintile of intake. Likewise, associations between vitamin C (2.3 95% CI 1.1, 3.6 per quintile *P*-trend < 0.001) and total carotene (1.7 95% CI 0.4, 2.9 per quintile *P*-trend = 0.009) and LEP (w/kg) were only observed in participants aged < 65 years. Similar trends were also found with FFMI and FFM/BMI, although there was no significant association between FFM/BMI and vitamin C in those < 65 years.

**Table 3:** Relative associations between indices of fat-free mass and leg explosive power calculated according to quantile of nutrient intake in 2570 females aged 18–79 years, stratified by age^a^

	All (*n* = 2570)		< 65 years (*n* = 2346)		≥ 65 years (*n* = 224)	
Quintile of nutrient intake	β (95% CI)	*P*-trend	β (95% CI)	*P*-trend	β (95% CI)	*P*-trend
FFM (%)						
Vitamin C (mg/d)	0.28 (0.1, 0.5)	< 0.01	0.30 (0.1, 0.5)	< 0.01	0.50 (− 0.5, 1.5)	0.32
Vitamin E (mg/d)	0.41 (0.2, 0.6)	< 0.01	0.41 (0.2, 0.7)	< 0.01	0.31 (− 0.7, 1.4)	0.55
Total carotene (µg/d)	0.14 (0.0, 0.3)	0.12	0.13 (− 0.1, 0.3)	0.16	0.63 (− 0.2, 1.5)	0.14
Retinol (µg/d)	0.00 (− 0.2, 0.2)	0.99	− 0.01 (− 0.2, 0.2)	0.92	0.32 (− 0.6, 1.3)	0.51
Protein (%E/d)	− 0.32 (− 0.5, − 0.2)	< 0.01	− 0.34 (− 0.5, − 0.2)	< 0.01	− 0.50 (− 1.5, 0.5)	0.34
LEP^b^ (w/kg)						
Vitamin C (mg/d)	2.14 (0.9, 3.3)	< 0.01	2.32 (1.1, 3.6)	< 0.01	− 1.06 (− 9.0, 6.9)	0.79
Vitamin E (mg/d)	0.66 (− 1.0, 2.3)	0.43	0.60 (− 1.2, 2.4)	0.51	− 0.76 (− 9.4, 7.8)	0.86
Total carotene (µg/d)	1.35 (0.1, 2.6)	0.03	1.67 (0.4, 2.9)	< 0.01	− 6.68 (− 15.0, 1.6)	0.11
Retinol (µg/d)	− 0.23 (− 1.5, 1.1)	0.73	− 0.39 (− 1.8, 1.0)	0.57	0.26 (− 8.8, 9.4)	0.95
Protein (%E/d)	− 1.18 (− 2.3, 0.0)	0.04	− 1.18 (− 2.4, 0.0)	0.05	− 2.64 (− 8.6, 3.3)	0.38
FFMI (kg/m^2^)						
Vitamin C (mg/d)	0.07 (0.0, 0.1)	< 0.01	0.08 (0.03, 0.12)	< 0.01	0.02 (− 0.3, 0.3)	0.91
Vitamin E (mg/d)	0.00 (− 0.05, 0.06)	0.85	− 0.01 (− 0.07, 0.05)	0.70	0.23 (− 0.1, 0.6)	0.17
Total carotene (µg/d)	0.06 (0.02, 0.1)	< 0.01	0.05 (0.01, 0.1)	0.02	0.22 (− 0.03, 0.46)	0.09
Retinol (µg/d)	− 0.03 (− 0.08, 0.02)	0.26	− 0.04 (− 0.1, 0.01)	0.11	0.13 (− 0.16, 0.43)	0.38
Protein (%E/d)	0.03 (− 0.01, 0.07)	0.15	0.03 (− 0.01, 0.1)	0.14	0.05 (− 0.23, 0.33)	0.73
FFM/BMI						
Vitamin C (mg/d)	0.00 (− 0.0, 0.01)	0.08	0.01 (− 0.00, 0.01)	0.11	0.02 (− 0.01, 0.05)	0.26
Vitamin E (mg/d)	0.01 (0.0, 0.02)	< 0.01	0.02 (0.01, 0.03)	< 0.01	− 0.00 (− 0.04, 0.04)	0.96
Total carotene (µg/d)	0.00 (− 0.0, 0.0)	0.49	0.00 (− 0.00, 0.01)	0.54	0.02 (− 0.01, 0.05)	0.24
Retinol (µg/d)	0.00 (− 0.0, 0.01)	0.23	0.00 (− 0.00, 0.01)	0.24	0.01 (− 0.02, 0.05)	0.43
Protein (%E/d)	− 0.02 (− 0.02, − 0.01)	< 0.01	− 0.02 (− 0.02, − 0.01)	< 0.01	− 0.02 (− 0.05, 0.02)	0.38

*FFM* fat free mass and *LEP* leg explosive power

^a^Values are adjusted beta coefficients (95% CI) per quantile of intake, *n* = 2570 (Quintiles for all ages and < 65 years and tertiles for those ≥ 65 year). Models were adjusted for age, physical activity, smoking status, energy intake and underreporting

^b^Subset analysis *n* = 1914 (< 65 years *n* = 1794; ≥ 65 years *n* = 120). Means were adjusted for age, physical activity, smoking status, energy intake, underreporting, menopausal status, hormone replacement therapy and height. Dietary variables are expressed per quintile for all participants and participants < 65 years and tertiles for participants ≥ 65 years

## Standardised Analyses

**Fig. 2:**
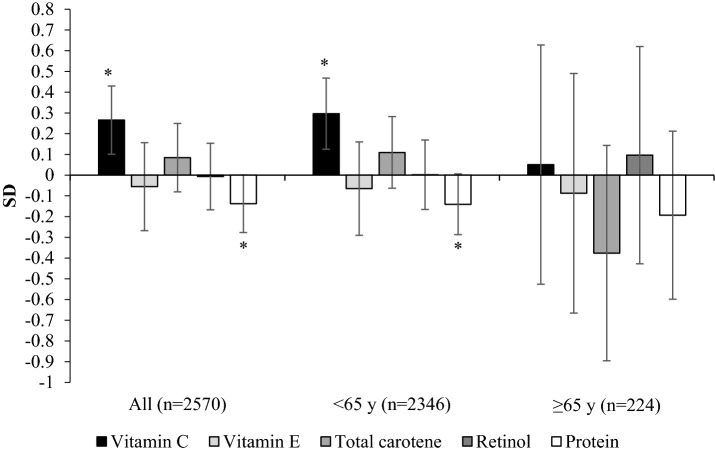
The relative associations of vitamin C, vitamin E, retinol, carotene and protein with leg explosive power in 1914 females aged 18–79 years, stratified by age^1^. Values represent the difference in standardised values of leg explosive power between participants in Q5 vs Q1 (T3-T1 for > 65 years sub-group) of intake with all nutrients included in the model. Values were also adjusted for age, physical activity, smoking status, energy intake, underreporting, height, menopausal status and use of hormone replacement therapy. * *P*-trend < 0.05

In standardised analyses, we compared the relative associations of vitamins C, E, retinol and total carotene with FFM% and LEP (Figs. 1, 2). Associations between vitamin C and FFM% and LEP were maintained after mutual adjustment for the other nutrients, including protein (FFM% 0.17 SD ± 0.07 *P*-trend = 0.013 and LEP 0.27 SD ± 0.08 *P*-trend = 0.004). A significant negative association with protein was observed for FFM% (− 0.18 SD ± 0.06 *P*-trend < 0.001), FFM_BMI_ − 0.27 ± 0.06 *P*-trend < 0.001 and LEP (− 0.14 SD ± 0.07 *P*-trend = 0.007) but the association with FFMI was not significant 0.05 SD ± 0.06 *P*-trend = 0.390. The results for vitamin E, not shown in the figures were: FFMI − 0.05 SD ± 0.08 *P*-trend = 0.487 and FFM_BMI_ 0.19 ± 0.09 *P*-trend = 0.025.

**Fig. 3:**
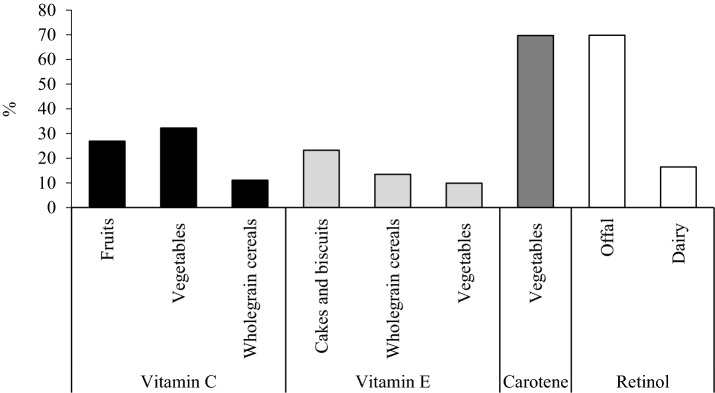
Foods that contributed to at least 10% of vitamin C, vitamin E, carotene and retinol intakes in 2570 females aged 18–79 years^1^. Values are the percentage contribution of individual foods to total nutrient intake. The main contributors to vegetable intakes were peppers, Brussels sprouts and broccoli for Vitamin C, avocado, mushrooms and spinach for Vitamin E and carrots and spinach for carotene. For Vitamin C the main contributors to fruit intakes were strawberries, oranges and grapefruit

Total vegetable intake was a significant source of vitamins C, E and carotene intake, however, the specific vegetables that contributed differed for each nutrient (Fig. 3). For vitamin C the main contributors were peppers, Brussels sprouts and broccoli, for vitamin E avocado, mushrooms and spinach, and for carotene, carrots and spinach. Fruit intake also contributed to vitamin C intake, and for vitamin E, the food groups cakes and biscuits and whole grain cereals contributed more to intake than vegetables.

## Discussion

Our findings of significant associations between higher intakes of vitamin C, total carotene and specific carotenoids and improved sarcopenic indices of skeletal muscle; FFM and LEP were found in a cohort of women with a wide age range (18–79 years). The scale of the associations for these vitamins ranged from 1.0 to 3.2% for indices of FFM and from 6.3 to 12.8% for LEP, comparing the highest and lowest intakes and adjusting for relevant covariates including age. This highlights the potential clinical importance of the findings as the yearly losses of FFM and strength range from 1%, for skeletal muscle mass, and 3% for grip strength, for people over the age of 50 years [29]. Moreover, using standardised analyses to compare the different vitamins and protein, the associations were greatest for vitamin C, despite mutual adjustment for all the nutrients investigated in our study, including protein intake. This research highlights the importance of dietary associations with muscle mass in individuals of all ages, supporting previous research that has shown that notable changes in skeletal muscle mass occur earlier in adult life (between 30 and 45 years of age) [30, 31].

The range of nutrients across quintiles of intake investigated in our study varied from 3.1 fold for vitamin E, and fourfold for vitamin C, to 4.6 fold for total carotene and to larger differences for retinol (10.4 fold). However, the associations with vitamin E intake were only found with FFM expressed either as a percentage or divided by BMI, and no associations were found with retinol.

Different foods contributed to intake of the nutrients investigated. Although vegetables were the most significant source, the specific vegetables contributing to these nutrients differed. The largest vegetable contributors to vitamin C were peppers, Brussels sprouts and broccoli. For carotene the major vegetable sources were carrots and spinach whereas vitamin E was supplied by avocado and mushrooms. Overall fruit was the greatest contributor to vitamin C intakes, and whole grain cereals were the greatest contributor to vitamin E intake. Our findings highlight the importance of eating a broad range of vegetables, fruits and whole grain cereal foods to achieve optimal intakes of vitamin C, carotenes and vitamin E.

## Comparison with Other Studies

The positive associations we found between higher intakes of vitamin C, carotenoids and vitamin E are, in the main, supported by the few previous human studies that investigated the relationships between intake or circulating concentrations of vitamin C, E or carotene and sarcopenic indices in cohorts of older people [11–14]. Dietary vitamin C was associated with measures of skeletal muscle function in the InCHIANTI Study, and in women only in the UK Hertfordshire Cohort Study (HCS) [11–14]. Circulating vitamin C and sarcopenic indices were only previously examined in elderly Japanese women where positive associations were found between measures of physical function but not FFM [13]. The only study investigating vitamin C intake with FFM found positive associations but only after 2.6 years of follow-up [11].

Circulating vitamin E was investigated in only three previous studies where plasma α-tocopherol was positively associated with measures of grip and knee strength, performance or decline in physical function. However, two studies found no associations between intake of vitamin E and either grip strength or function [12, 14, 17, 32]. Our finding of a positive relationship with dietary vitamin E and certain indices of fat-free mass further supports the relevance of vitamin E to skeletal muscle health.

For carotenoids, two previous studies found associations with either circulating β or total carotene and greater muscle strength or physical activity, with only one other study finding positive associations between higher intakes of carotene and strength or physical activity, in women [12, 14–19]. Our findings extend the previous research by identifying that the full range of carotenoids also have importance for skeletal muscle health.

The lack of association with retinol intake and sarcopenic indices found in our study contrasts with the only other human study that found greater intakes of retinol were significantly related to loss of appendicular lean mass [11].

In recent studies, relating intakes of either vitamin C, E, or circulating vitamin E, to sarcopenia no associations were found [33–35] although greater prevalence of frailty was associated with either lower concentrations of carotenoids, retinol or α-tocopherol, in a further three studies [36–39].

The reason for the lack of association between the vitamins in our study and grip strength, compared with the strong associations with LEP are not clear but may be due to LEP being a direct measure of lower limb strength and power which may be more sensitive to the effects of diet [29].

The lack of association between the vitamins and CRP in our study may be due to the relatively high intakes of vitamins, coupled with the relatively low blood concentrations of hsCRP in this population.

Although we also found smaller associations with the sarcopenic indices and protein intake than the vitamins in our standardised analyses, other studies have found protein is important for skeletal muscle. However, there has been variability in effectiveness of supplementation with protein in intervention studies, and protein intake in our study was higher, 1.3 g protein/kg/d, than in other previous studies which may explain our findings [10, 40]. However, our findings relating to protein intake require further exploration. Nevertheless, our findings suggest that consuming sufficient antioxidant vitamins in addition to protein is important.

Despite the few previous studies, to our knowledge, our study is the first to investigate dietary intakes of vitamin C, E, A (retinol), individual carotenoids and protein, as well as circulating CRP with the full range of sarcopenic indices in both younger and older women.

## Physiological Mechanisms

There is clear mechanistic relevance of the antioxidant vitamins on skeletal muscle health during aging which also supports our observational findings. Supplementation of lycopene, β-carotene or mixed antioxidant vitamins in animal experiments either had positive effects on muscle force or physical activity or attenuated oxidative stress or loss of skeletal muscle [41]. Vitamin E also had protective effects on exercise-induced oxidative damage in both young and older adults [7], and vitamin C had protective effects on oxidative biomarkers, inflammatory cytokines and CRP [42]. Beyond roles as an antioxidant and anti-inflammatory agent vitamin C is also integral to synthesis of collagen and of carnitine which is important for the metabolism of long-chain fatty acids during physical activity [8].

## Deficiency of Vitamins and Supplementation

Although the significant associations between intake of vitamins C, E and carotenoids were found across the usual range of consumption, the women in the lowest quintile of vitamin C intake consumed less than the average requirement for vitamin C intake of 80 mg/d [43]. The prevalence of low intakes of vitamin C, or circulating concentrations of vitamin C indicative of scurvy, is high in vulnerable older populations living in community and residential care ~ 40% [3, 44]. Moreover, consumption of 5 or more portions of fruits and vegetables a day is only 19% of those over the age of 75 years in the UK [45].

Whilst supraphysiological doses of vitamin C could be used to rectify low dietary intakes in older vulnerable groups these need to be used with caution due to the detrimental, prooxidant, effects of long-term high doses of vitamin C (> 500 mg per day) compared the concentrations found naturally in foods [46]. There is also debate surrounding potential negative effects of supraphysiological doses of vitamin C on skeletal muscle function in athletes [47].

Our research has found that intake of antioxidant vitamins within the normal dietary range has important effects on sarcopenic indices, in the whole cohort and stratified analysis showed associations in younger women, provided by a different food groups. Given the shortfall in intakes of the antioxidant vitamins it is important to encourage greater intakes of the foods that supply them; namely vegetables, fruits and whole grain cereal foods, in addition protein for maintaining skeletal muscle health in populations of all ages. This is particularly important for vulnerable older people in the community and in residential care.

Strengths of this study include comprehensive measurements of diet in a well characterised large cohort which allowed us to examine for the first time associations between an extensive range of sarcopenic indices, including DXA-measured FFM, LEP and grip strength and a range of antioxidant vitamins, including all the carotenoids. We were also able to undertake a comparative analysis of the different nutrients allowing us to examine the independent associations with intake of each vitamin and protein. Limitations include the small number of women aged over 65 an imbalance between younger and older women in our cohort which future studies should address. In addition, this study was cross-sectional which limits inference for causation. We also did not account for the nutrients supplied by food supplements, however, our analyses indicate the importance of intake of vitamins supplied by diet alone. A further limitation is that we did not have directly measured physical activity, however, we used validated questionnaires, which although less precise than objective measures, do distinguish across the range of activity levels in individuals [28].

Although we were able to determine what dietary intakes are relevant for skeletal muscle health we lacked data on dietary biomarkers meaning we were unable to confirm our findings with blood or urinary levels.

## Conclusion

For the first time in a free-living population, our research shows that higher dietary intake of the antioxidant vitamins, particularly vitamin C, could be protective for both loss of skeletal muscle mass and power during aging, and have relevance for treatment and prevention of frailty and sarcopenia in women. It was notable that the observed associations were identified in women younger than 65 years. Our findings provide further encouragement for intervention trials as well as for following the healthy eating guidelines for protection of skeletal muscle mass and power in people of all ages.

## Electronic supplementary material

Below is the link to the electronic supplementary material.

(ppt 169 kb)
