# Supplementary material for: Cross-Sectional Associations Between Dietary Antioxidant Vitamins C, E and Carotenoid Intakes and Sarcopenic Indices in Women Aged 18–79 Years
Source: Calcif Tissue Int. 2019 Dec 7;106(4):331–42. doi: 10.1007/s00223-019-00641-x (PMC7072069; doi:10.1007/s00223-019-00641-x)
Supplement: Supplementary file 1 — (ppt 169 kb) [file 223_2019_641_MOESM1_ESM.ppt]

## Slide 1
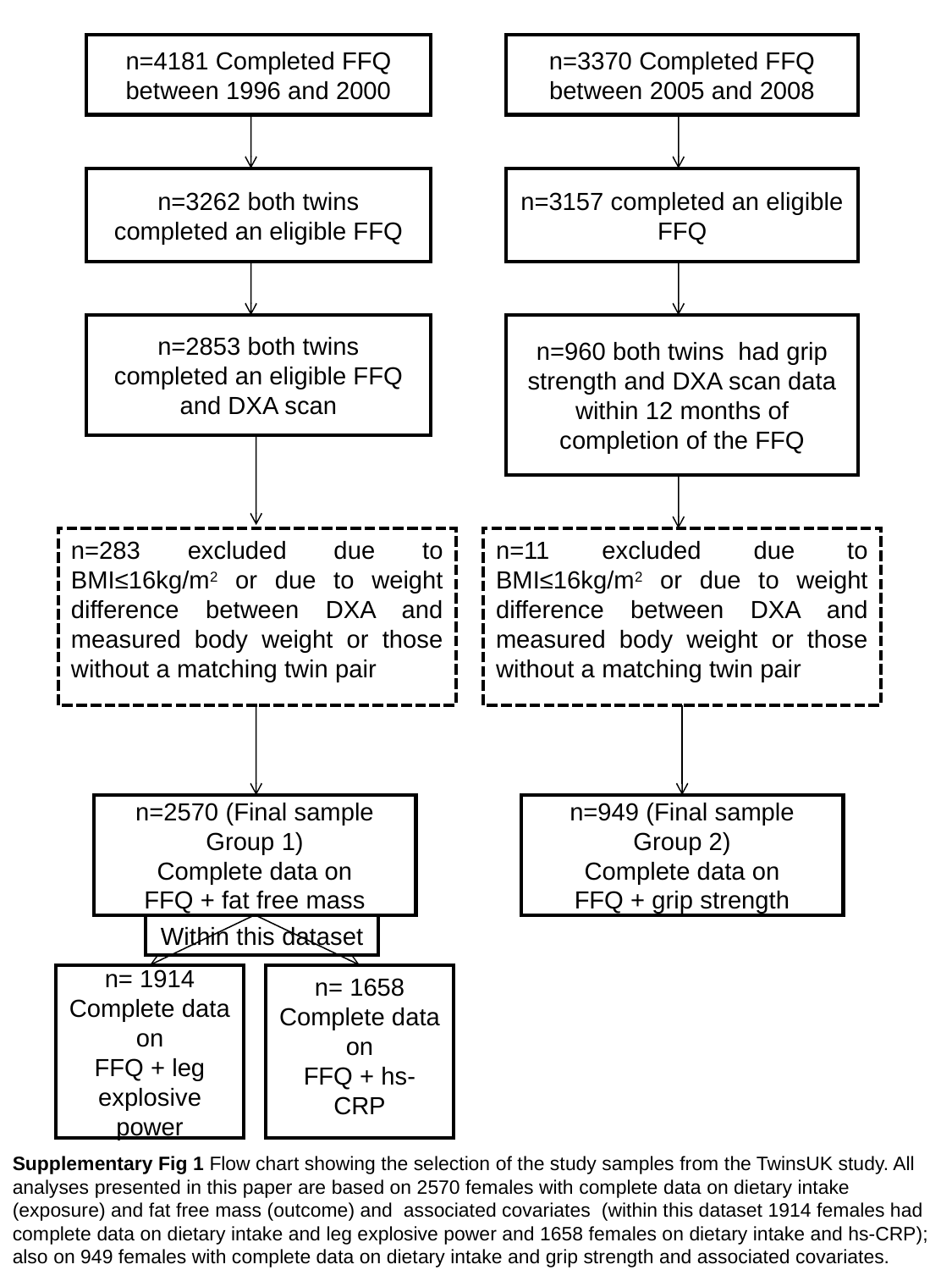

n=4181 Completed FFQ between 1996 and 2000
n=3370 Completed FFQ between 2005 and 2008
n=3262 both twins completed an eligible FFQ
n=3157 completed an eligible FFQ
n=2853 both twins completed an eligible FFQ and DXA scan
n=960 both twins had grip strength and DXA scan data within 12 months of completion of the FFQ
n=283 excluded due to BMI≤16kg/m2 or due to weight difference between DXA and measured body weight or those without a matching twin pair
n=11 excluded due to BMI≤16kg/m2 or due to weight difference between DXA and measured body weight or those without a matching twin pair
n=2570 (Final sample Group 1)
Complete data on
FFQ + fat free mass
n=949 (Final sample Group 2)
Complete data on
FFQ + grip strength
Within this dataset
n= 1914
Complete data on
FFQ + leg explosive power
n= 1658
Complete data on
FFQ + hs-CRP
Supplementary Fig 1 Flow chart showing the selection of the study samples from the TwinsUK study. All analyses presented in this paper are based on 2570 females with complete data on dietary intake (exposure) and fat free mass (outcome) and associated covariates (within this dataset 1914 females had complete data on dietary intake and leg explosive power and 1658 females on dietary intake and hs-CRP); also on 949 females with complete data on dietary intake and grip strength and associated covariates.
